# Analysis of *Staphylococcus aureus* wall teichoic acid glycoepitopes by Fourier Transform Infrared Spectroscopy provides novel insights into the staphylococcal glycocode

**DOI:** 10.1038/s41598-018-20222-6

**Published:** 2018-01-30

**Authors:** Tom Grunert, Dijana Jovanovic, Wanchat Sirisarn, Sophia Johler, Christopher Weidenmaier, Monika Ehling-Schulz, Guoqing Xia

**Affiliations:** 1 Functional Microbiology, Institute of Microbiology, Department of Pathobiology, University of Veterinary Medicine Vienna, Vienna, Austria; 2 Division of Infection, Immunity and Respiratory Medicine, School of Biological Sciences Faculty of Biology, Medicine and Health, Manchester Academic Health Science Centre, University of Manchester, Manchester, M13 9PT United Kingdom; 3 Institute for Food Safety and Hygiene, Vetsuisse Faculty University of Zurich, Zurich, Switzerland; 4 Interfaculty Institute for Microbiology and Infection Medicine Tübingen, University of Tübingen and German Center for Infection Research, Tübingen, Germany

## Abstract

Surface carbohydrate moieties are essential for bacterial communication, phage-bacteria and host-pathogen interaction. Most *Staphylococcus aureus* produce polyribitolphosphate type Wall teichoic acids (WTAs) substituted with α- and/or β-O-linked N-acetyl-glucosamine (α-/β-O-GlcNAc) residues. GlcNAc modifications have attracted particular interest, as they were shown to govern staphylococcal adhesion to host cells, to promote phage susceptibility conferring beta-lactam resistance and are an important target for antimicrobial agents and vaccines. However, there is a lack of rapid, reliable, and convenient methods to detect and quantify these sugar residues. Whole cell Fourier transform infrared (FTIR) spectroscopy could meet these demands and was employed to analyse WTAs and WTA glycosylation in *S. aureus*. Using *S. aureus* mutants, we found that a complete loss of WTA expression resulted in strong FTIR spectral perturbations mainly related to carbohydrates and phosphorus-containing molecules. We could demonstrate that α- or β-O-GlcNAc WTA substituents can be clearly differentiated by chemometrically assisted FTIR spectroscopy. Our results suggest that whole cell FTIR spectroscopy represents a powerful and reliable method for large scale analysis of WTA glycosylation, thus opening up a complete new range of options for deciphering the staphylococcal pathogenesis related glycocode.

## Introduction

The *Staphylococcus aureus* cell envelope comprises a variety of secondary surface glycopolymers including capsule polysaccharide (CP), poly-β(1–6)-N-acetylglucosamine (PNAG), and wall teichoic acid (WTA)^1^. These cell surface glycostructures are known to affect bacterial interactions with hosts in multiple ways and are important targets for antimicrobial agents and vaccines^2,3^. In particular, WTAs are highly abundant surface polymers that play important roles in *S. aureus* physiology and pathogenesis such as cell division, phage susceptibility, biofilm formation, interaction with host cells and complement activation^4–6^. One of the main sources of structural diversity in polysaccharides is the type of glycosidic linkage. Recently, the WTA glycosylation pathways in *S. aureus* have been elucidated, involving two glycosyltransferase TarM and TarS that attach N-Acetylglucosamine (GlcNAc) to the WTA main chain via O-linkage in α- and β-anomeric configuration, respectively^7,8^. α-O-GlcNAc modified WTA plays an important role as phage receptor and promotes the transduction of staphylococcal DNA via horizontal gene transfer (HGT), whereas, a lack in β-O-GlcNAc modification sensitize methicillin resistant *S. aureus* (MRSA) strains to β-lactams^6,7,9^. This variable structure of glycosylated WTA constitutes a specific glycocode and it remains elusive why the presence or absence of both anomeric linkage types differ among clinical isolates.

Although, the presence of the *tarM or tarS* genes can be easily detected by PCR, to date, detection and quantification of WTA GlcNAc residues remains challenging. Often, purified WTA samples and nuclear magnetic resonance (NMR) spectroscopy are required, rendering detection and quantification of WTA GlcNAc residues both laborious and time consuming. Therefore a rapid, cost-effective, and a reliable method is urgently needed to analyse WTA α- and β-O-GlcNAc modifications in *S. aureus*.

Whole cell molecular fingerprinting by Fourier transform infrared (FTIR) spectroscopy represents a particularly promising approach. This user-friendly, high-throughput tool has not only been successfully implemented in bacterial identification and subtyping, but also allows users to follow phenotypic responses^10–12^. In *S. aureus*, FTIR spectroscopy was applied to analyse the charge of extracted WTA in relation to its alanine substitutions and the phosphate group in its main chain^13^. Recently, we were able to introduce chemometric-assisted FTIR spectroscopy as a highly discriminatory and reliable tool in the differentiation of *S. aureus* capsular serotypes CP5, CP8, and non-typeable (NT)^14^. We further demonstrated that highly discriminatory subtyping of *S. aureus* by FTIR spectroscopy primarily relies on the differential expression of capsular serotypes and/or additional surface glycopolymers such as WTA, peptidoglycan, and lipoteichoic acid^15^.

In this study, we aim to evaluate the suitability of FTIR spectroscopy as a tool for discrimination of WTA glycoepitopes, particularly between α-O-GlcNAc and β-O-GlcNAc modified WTA.

## Results

### Complete loss of wall teichoic acids causes strong perturbations in whole cell FTIR spectra of *S. aureus*

**Figure 1:**
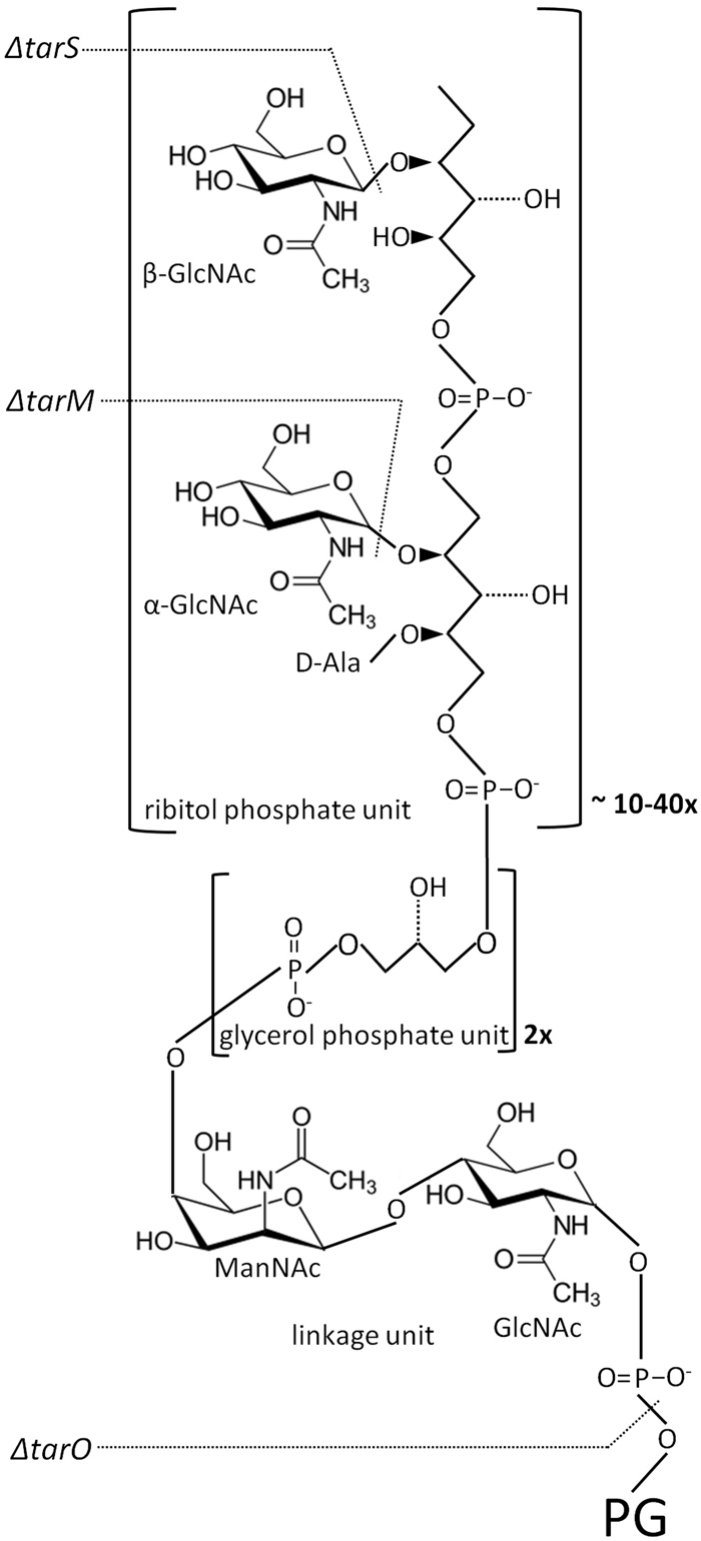
Schematic *S. aureus* WTA structure. *S. aureus* WTA consists of an ManNAc-GlcNAc disaccharide linkage unit connected to the peptidoglycan layer in the bacterial cell wall, two glycerol phosphate units and a long chain of ribitol phosphate repeating units substituted with α- or β-GlcNAc and D-alanine (adapted from Kurokawa *et al*.^23^). Structural changes by mutations of Δ*tarS*, Δ*tarM*, or Δ*tarO* are indicated. GlcNAc, N-acetylglucosamine; ManNAc, N-acetylmannoseamine; D-Ala, D-alanine; PG, peptidoglycan.

**Figure 2:**
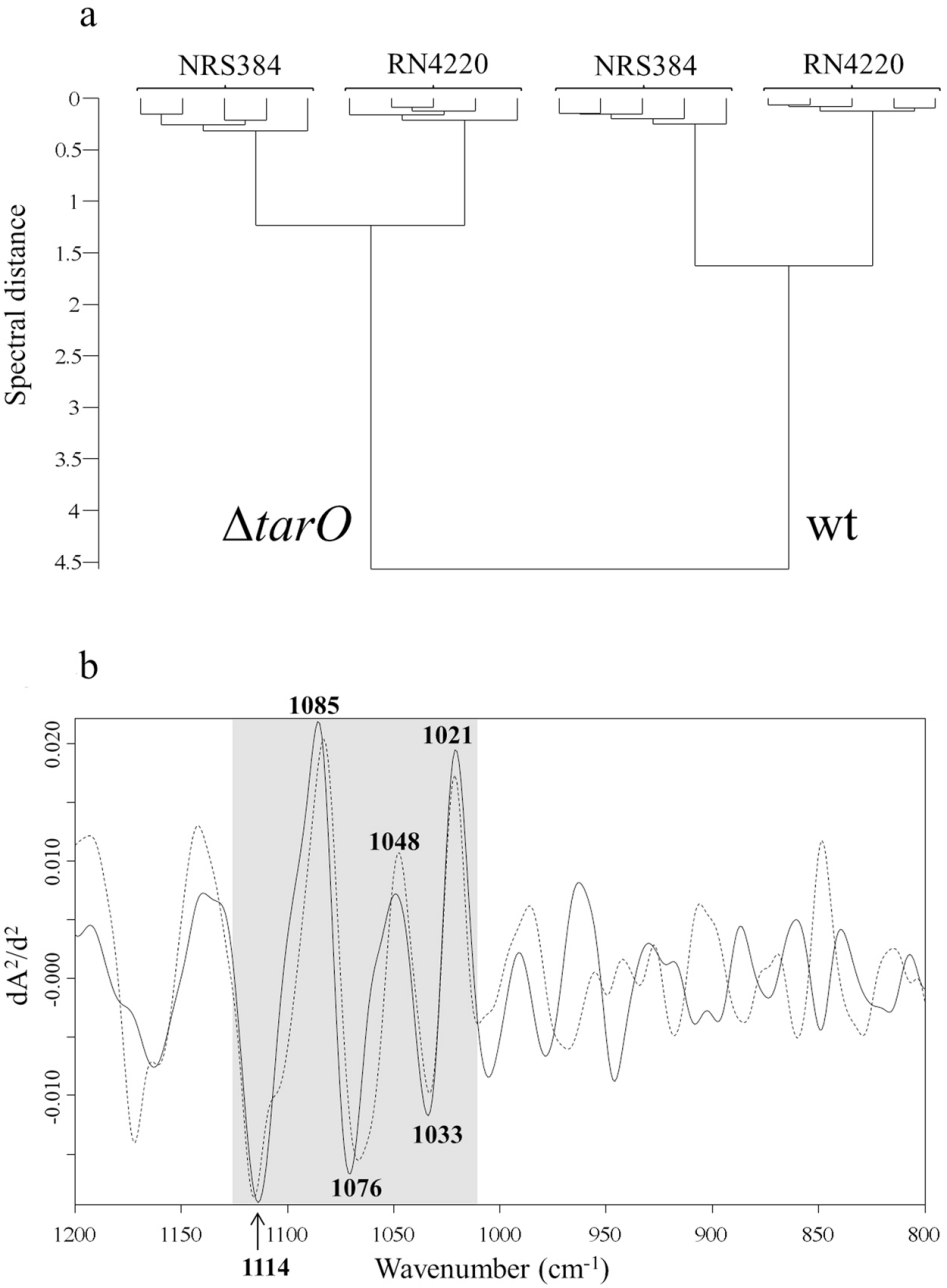
Spectral impact of lack of *S. aureus* WTA. (**a**) FTIR spectroscopy-based dendrogram (HCA) of intact wild type (RN4220, NRS384) and isogenic ∆*tarO* mutant strains, each comprising measurements at five different days. (**b**) Subtraction spectra were generated from second derivative, vector-normalized, average FTIR spectra by subtracting spectra of ∆*tarO* from those of the wild type (RN4220, solid; NRS384, dashed). Most pronounced differences for both strains (RN4220, NRS384) were observed in the spectral range of 1120–1010 cm^−1^, which can be assigned to carbohydrate-associated vibrations and phosphodiester functional groups (see also Table 1).

Whole cell FTIR spectra of wild type *S. aureus* strains RN4220 and NRS384 (also known as USA300) were compared to those of isogenic Δ*tarO* mutants. As shown in in Fig. 1, the mutation of the Δ*tarO* gene results in the complete loss of *S. aureus* WTA. Growth experiments and FTIR measurements were carried out five times independently. The original, average absorbance spectra over the whole spectral range (4000–500 cm^−1^) of strain RN4220 and their mutants can be found as Supplementary Fig. S1. 2^nd^-derivatives were calculated from the original absorbance spectra to increase spectral resolution and to minimize contributions of baseline shifts followed by vector normalization to adjust for variations in bacterial biomass among different sample preparation. An exploratory, hierarchical cluster analysis (HCA) was carried out to detect inherent spectral differences between wild type strains and strains lacking WTA using the spectral range of 1200–800 cm^−1^, previously described to be highly discriminatory for *S. aureus* surface glycostructures. Both Δ*tarO* mutants clustered separately from their isogenic wild type strains (Fig. 2a). Subtractive spectral analysis was performed to obtain relevant information of spectral differences by subtracting 2^nd^ derivative/vector normalized spectra of ∆*tarO* from those of the corresponding wild types (RN4220, NRS384). The complete loss of WTA synthesis caused highly discriminatory spectral changes at wavenumbers between 1125 cm^−1^ and 1010 cm^−1^, as shown by subtractive spectral analysis in Fig. 2b. Within this spectral range, a series of prominent differences at 1114 cm^−1^, 1085 cm^−1^, 1076 cm^−1^, 1048 cm^−1^, 1033 cm^−1^ and 1021 cm^−1^ could be detected.

### Spectral differentiation of *S. aureus* α- and β-O-GlcNAc WTA substituents

**Figure 3:**
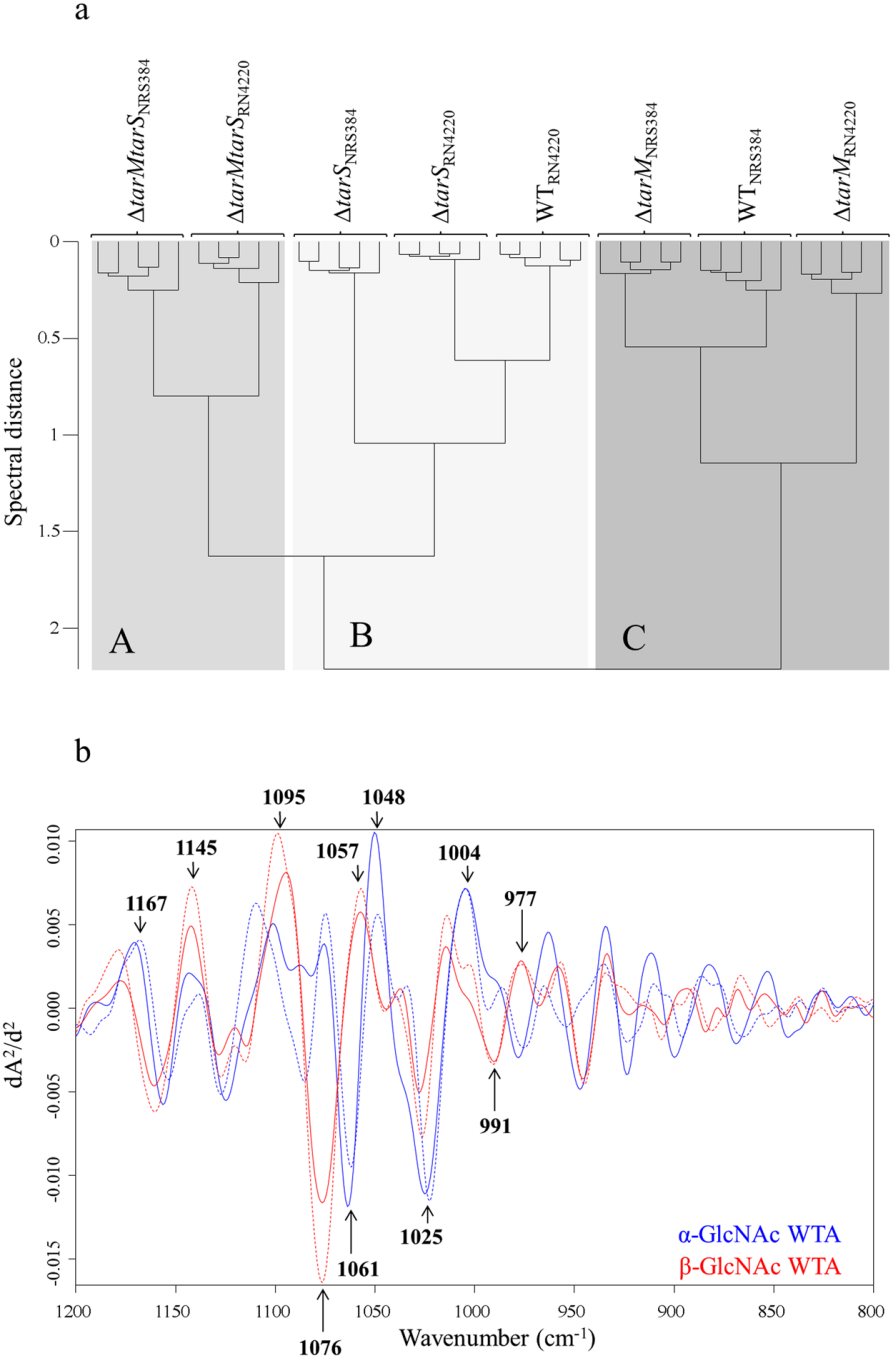
Spectral differentiation of *S. aureus* α- and β-O-GlcNAc WTA substituents. (**a**) FTIR spectroscopy-based dendrogram (HCA) of intact wild type (RN4220, NRS384) and isogenic ∆*tarM*, ∆*tarS* and ∆*tarMS* mutants, each comprising duplicate measurements at five different days. (**b**) Spectral assignments of α-GlcNAc and β-GlcNAc modified RboP WTA. Subtraction spectra were generated using second derivative, vector-normalized FTIR average spectra of ∆*tarM* and ∆*tarS* strains and the respective isogenic double mutant ∆*tarMS* of RN4220 (solid) and NRS384 (dashed). Spectra from ∆*tarM*∆*tarS* mutant strains were substracted from either (1) ∆*tarM* exhibiting the glycosyltransferase TarS expression corresponding to the β-GlcNAc WTA (red) or (2) ∆*tarS* exhibiting the glycosyltransferase TarM expression corresponding to the α-GlcNAc WTA (blue). Main spectral differences in the modification of RboP WTA with α-GlcNAc and β-GlcNAc substituents are in the spectral range of 1175–970 cm^−1^, which can be assigned to vibrations of carbohydrates and their glyosidic linkages.

Various mutant strains derived from wild type *S. aureus* strain RN4220 and NRS384 were subjected to whole cell FTIR spectroscopic analysis. These include Δ*tarM* mutants deficient in WTA α-O-GlcNAc, Δ*tarS* mutants lacking WTA β-O-GlcNAc residues and double mutants (Δ*tarM*Δ*tarS*) without any GlcNAc residues on WTA (Fig. 1). FTIR-HCA (Fig. 3a) revealed distinct clustering (A-C) for WTA glycosyltransferase mutants Δ*tarM*, Δ*tarS*, and Δ*tarM*Δ*tarS*, respectively. Interestingly, the wild type strain RN4220 was grouped into cluster B, which includes all Δ*tarS* mutants deficient in WTA β-O-GlcNAc. This is in good agreement with previous observations that 90% GlcNAc residues on WTA have α-linkage in RN4220. Of note, wild type NRS384 was grouped into cluster C, which includes all Δ*tarM* mutants lacking WTA α-O-GlcNAc suggesting that WTA GlcNAc residues in NRS384 most probably exhibit β-configuration. Data available at transcriptional level point to a constituently higher expression of *tarS* (WTA β-O-GlcNAc) compared to *tarM*, which largely depends on growth conditions^16^. Subtraction spectral analysis supports this assumption showing differences in intensities of several vibrational frequencies for the α- and β-glycosidic substitution of GlcNAc residues (Fig. 3b): 1167 cm^−1^, 1145 cm^−1^, 1095 cm^−1^, 1076 cm^−1^, 1061 cm^−1^, 1057 cm^−1^, 1048 cm^−1^, 1025 cm^−1^, 1004 cm^−1^, 991 cm^−1^ and 977 cm^−1^. However, this cannot be finally validated, since the exact amount and relation of α- and β-O-GlcNAc for strain NRS384 are currently unknown.

**Figure 4:**
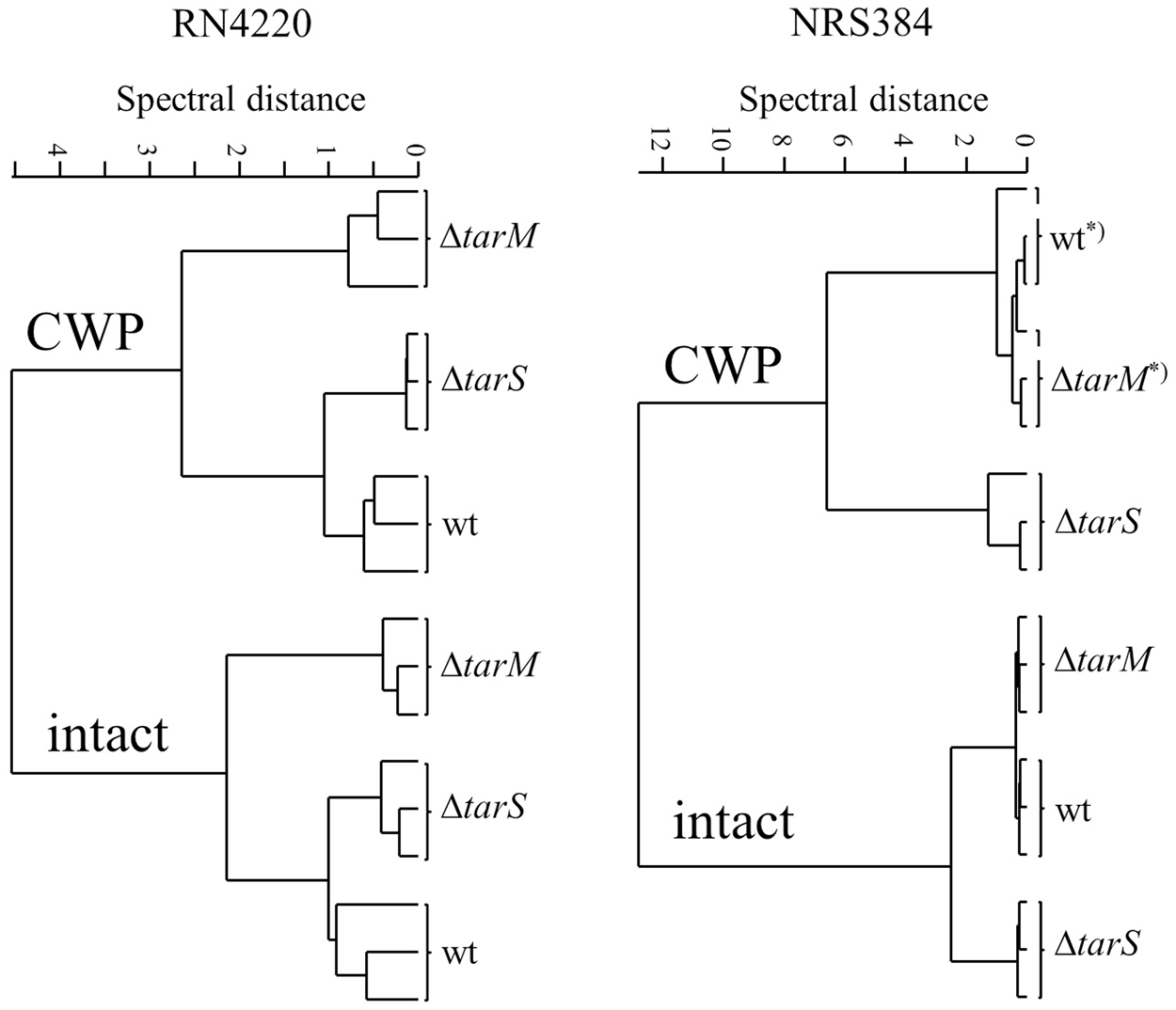
Spectral comparison between intact bacterial cells and their cell wall preparation. FTIR spectroscopy-based dendrogram of RN4220 and NRS384 WT and their corresponding mutant strains ∆*tarM* and ∆*tarS* recorded from intact bacterial cells and after cell wall preparation (CWP). Spectra derived from independent growth experiments and subsequent cell wall preparations at three different days. Since a consistent clustering of wild type and mutant strains derived from cell wall preparations and intact bacteria cells was achieved it can be assumed that major discriminatory components are related to the bacterial surface. *Several cell wall sample preparations steps result in a higher technical variation, which interferes differential clustering of minor spectral differences.

In order to evaluate if these differences obtained by FTIR spectroscopy can be particularly assigned to changes at the cellular surface and not to possible changes in the entire cellular composition of the bacteria, spectra of cell wall preparations were compared to spectra obtained from intact bacterial cells using HCA (Fig. 4). Since spectra derived from cell wall preparations of wild type, Δ*tarM* and Δ*tarS* mutants from each strain background (RN4220, NRS384) clustered consistent with spectra from intact bacterial cells, it can be assumed that the major discriminatory components are related to the bacterial surface. Thus, spectral changes due to α/β-O-GlcNAc modifications of RboP WTA can be characterized directly from intact *S. aureus* cells in a significantly faster and less-laborious manner.

**Figure 5:**
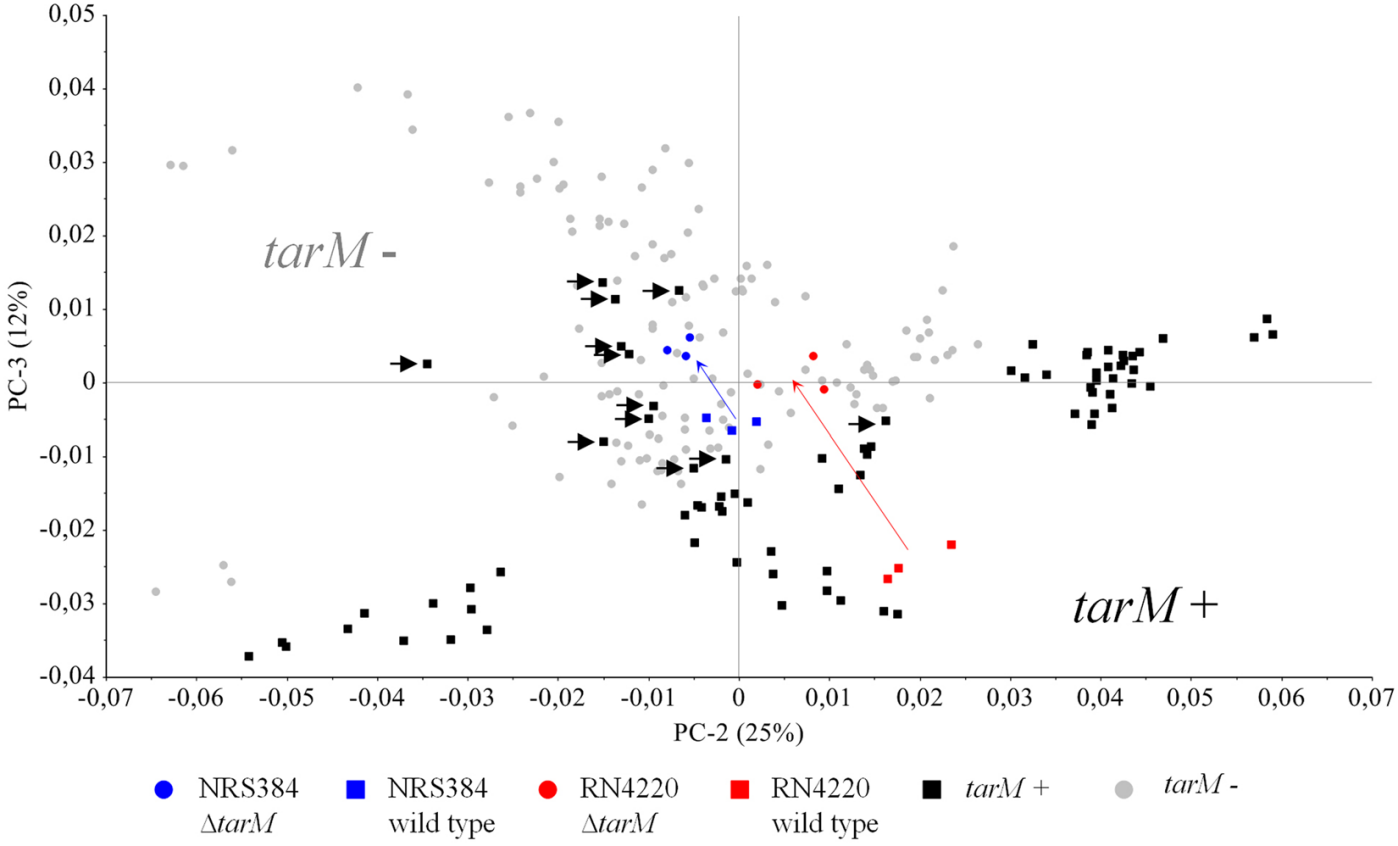
Spectral discrimination of α-O-GlcNAc glycoepitopes based on the presence of the *tarM* gene. PCA was performed from triplicate spectra of *tarM* positive and *tarM* negative strains from a diverse strain collection (n = 70) including various CCs. PC2 was plotted against PC3. PCA shows a distinct separation of the *tarM* positive and *tarM* negative strains, except the triplicate measurements of four strains (incl. NRS384 wild type) highlighted with an arrow. The *tarM* mutant strains of NRS384 and RN4220 were located in the *tarM* negative cluster, whereas the RN4220 wild type strain known for its high α-GlcNAc WTA content is located in the *tarM* positive cluster.

Reliable prediction of the expression ratio of α-O-GlcNAc and β-O-GlcNAc glycoepitopes by FTIR spectroscopy based on a single measurement of intact bacterial cells requires a sufficient amount of characterized strains. This would allow for the establishment of a supervised chemometric algorithm as shown previously for artificial neural network -assisted discrimination of *S. aureus* CP expression^14^. Due to the fact that expression data of the WTA α/β-O-GlcNAc –ratio of individual strains are not available and the *tarS* gene exits in every sequenced *S. aureus* genome, we focused on the correlation between the presence of the *tarM* gene and the signal signature of WTA α-O-GlcNAc glycoepitopes in the FTIR spectra. A well-characterized set of 70 *S. aureus* strains from human, animal, and food sources, which includes clonal complexes (CC) commonly isolated from asymptomatic and infected humans (CC5, CC8, CC30, and CC45) and animals (CC705,CC398) was used to determine the presence of the *tarM* gene. A total of 26 out of 70 strains (37.1%) were tested positive for *tarM*. Of note, while all tested CC705 strains are *tarM* positive, all tested CC398, CC5 and CC45 strains are *tarM* negative. CC30 and CC8 strains were found to be predominantly *tarM* positive, 70% and 60%, respectively (Suppl. 2). Our data also suggest that the presence of α-O-GlcNAc WTA may additionally contribute to the discrimination of *S. aureus* strains by FTIR spectroscopy, which is primarily based on CP expression (Suppl. 2). In particular, the *tarM* negative CC45 and *tarM* positive CC705 strains formed individual subclusters (A1 and A2) within the main cluster A of CP8 expressing strains. In addition, subcluster B2 only comprised *tarM* positive strains (n = 6) derived from different CC (CC15, CC8, CC22, CC30). Moreover, a principal component analysis (PCA) of FTIR spectra from this data set was carried out to correlate the inherent spectral data structures to the presence or absence of the *tarM* gene in a specific strain. PCA, an unsupervised multivariate data analysis technique, reduces the large spectral data space to several principal components (PCs) in an unbiased manner. As shown in Fig. 5, *tarM* negative and *tarM* positive strains can be mainly discriminated from each other by using the PC 2 (25%) and 3 (12%), while PC 1 discriminates mainly according to the CP expression of each strain. Each PC explains a certain percentage of the total variation in the original dataset and each sample has a score on it, which reflects the location of the sample along the PC. Thus, proximate samples at the scores plot are more similar to each other by forming a cluster. As expected, both *tarM* mutant strains (derived from either NRS384 or RN4220) were located in the *tarM* negative cluster, whereas the RN4220 wild type strain known to express 90% GlcNAc residues on WTA with the α-glycosidic linkage type was found in the *tarM*-positive strain cluster. However, triplicate measurements of four strains harbouring the *tarM* gene (all belong to CC8; highlighted with an arrow) and the NRS384 wild type (CC8) strain were located in the *tarM*-negative cluster. It can be assumed that these strains either do not or only marginally produce amounts of β-GlcNAc WTA. Further investigations with additional characterized WTA α/β-O-GlcNAc strains are needed to set up a functional, reliable supervised algorithm which can quantify the *S. aureus* α/β-O-GlcNAc WTA content or ratio.

## Discussion

In this study, we demonstrate that whole cell FTIR spectroscopy can be used to detect changes in *S. aureus* WTA glycoepitope composition. We focused on spectral bands in the wavenumber range of 1200–800 cm^−1^, known to be highly discriminatory for *S. aureus* capsule polysaccharide typing and subspecies differentiation^14,15^. A complete loss of *S. aureus* WTA expression (Δ*tarO* mutant) resulted in highly discriminatory changes within this spectral region, which had been previously assigned to various functional groups: (i) C-OH *str*, C-O-C *str*, Ring *str* vib of carbohydrates and (ii) P = O symmetric stretching of -PO_2_^−^ (Table 1). These assignments can be structurally correlated to the loss of carbohydrates and phosphodiester groups in the WTA of the Δ*tarO* mutant strain (Fig. 1).

**Table 1:** Assignment of bands of FTIR spectra mentioned in this report.

Frequency (cm^-1^)	Assignment	References
1200–900	C-OH stretching modes and C-O-C, C-O ring vibrations of carbohydrates in the bacterial cell wall	^24–32^
1175–1140	Differences in glycosidic linkage configuration	^19^
1160–1130 1120–1020	C-O *str* of C-O-C glycosidic linkage C-O-C *str* of carbohydrates	^17,31^
1085	P=O *sym str* of >PO_2_^−^	^25,28,30,31^
1080–1060	Ring *str* vib of carbohydrates	^31^
1080–1000	C-OH *str* of carbohydrates	^31^
1000–970	Differences in glycosidic linkage configuration	^19^
999–965	C-O *str* in C-O-C glycosidic linkages	^18^

*Str*, stretching; *sym*, symmetrical; *vib*, vibration.

The clustering of the glycosyltransferase Δ*tarM* and Δ*tarS* mutant as well as double Δ*tarM*Δ*tarS* mutant strains showed the high sensitivity of FTIR spectroscopy to specifically detect α- and β-GlcNAc substitutes of WTA in intact *S. aureus* cells. We could demonstrate that these spectral signatures are originated from the bacterial surface comparing spectra of cell wall preparations to spectra obtained from intact bacterial cells. Vibrational spectroscopy can detect not only carbohydrate molecule-specific bands but also bands interfering with the glycosidic linkage^17–19^. We could previously link a highly discriminatory spectral band at 834 cm^−1^ to a different α- and β-glycosidic linkage for N-acetyl-D-fucosamine (D-FucNAc) of *S. aureus* CP8 and CP5 capsular polysaccharides, respectively^14^. For differences of α- and β-O-GlcNAc WTA, the spectral range of 1175–970 cm^−1^ showed several different bands, which predominantly arise from (i) C-OH *str*, C-O-C *str*, Ring *str* vib of carbohydrates, and (ii) differences in the glycosidic linkage type, and are suggested to be affected by the removal of α- and β-GlcNAc WTA (Fig. 1, Table 1). Thus, they might be helpful to predict the presence or even the ratio of α-O-GlcNAc and β-O-GlcNAc WTA in the infrared spectrum of *S. aureus*. We found first indications for this, since the RN4220 and NRS384 wild type strains cluster either next to the Δ*tarS* or Δ*tarM* mutant strains. Moreover, PCA analysis could roughly correlate FTIR spectral patterns to the presence or absence of the *tarM* gene in *S. aureus*.

Recently, it was shown that in contrast to *tarS* which is present in almost all *S. aureus* genomes*, tarM* is strain specific and missing in certain CCs (CC398, CC5, CC45)^20^. We could confirm the lack of *tarM* for these CCs using a larger number of strains. We were also able to show that all tested CC705 (former CC151) isolates, which is very common among bovine mastitis, does harbour the *tarM* gene. Interestingly, some CC8 and CC30 strains turned out to be lacking the *tarM* gene. This supports the previously raised hypothesis that *tarM*-dependent WTA (α-glycosidic substitution) could play a role in host tropism^3^. We found 36.1% of all tested strains were *tarM* positive, which is in good agreement with the previously published observation for nasal isolates (35.7%)^20^.

Our data suggest that whole cell FTIR spectroscopy is a promising approach to detect WTA GlcNAc epitopes. However, current limitations in the availability of strains, which are well characterized for the presence of WTA α- and β-GlcNAc residues, impair accurate detection by FTIR spectroscopy. Thus, to further explore the potential of FTIR spectroscopy and to analyse the abundance of α- and β-GlcNAc modified WTA, more strains serving as reference have to be analysed for their specific glycosidic linkage type by specific immunobased detection and/or NMR spectroscopy. This would enable the establishment of FTIR spectroscopy as a rapid phenotyping method to decipher the role of surface carbohydrate moieties important for bio-communication to further decipher the *S. aureus* glycocode. These may include, but are not limited to, the carbohydrate structures contributing to *S. aureus* host specificity/tropism, the glycostructural dynamics due to time-dependent monitoring across various phases of growth and the dynamic arrangement due to exposure to host cells. In particular, as WTA β-GlcNAc residues are specifically required for the beta-lactam resistance in *S. aureus*, a typing scheme including whole cell FTIR spectroscopy would be more advantageous to predict the resistance phenotype when compared to conventional PCR based genotyping or more recent typing by Matrix Assisted Laser Desorption Ionization-Time of Flight Mass Spectrometry (MALDI-TOF-MS), as these approaches could neither provide the configuration nor the abundance of the WTA glycoepitopes. As it is appreciated that bacterial cell surface structure such as lipopolysaccharides (LPS), CP, PNAG, glycoproteins and WTAs play crucial roles in bacterial pathogenesis, antibiotic resistance, host-pathogen interaction and antibiotic resistance, clearly, further development of whole cell FTIR spectroscopy will enable efficient and specific detection and quantification of bacterial cell surface glycostructures of important diagnostic value, help developing novel specific, fast, convenient, phenotyping and diagnostics tools for detecting bacterial infections.

## Conclusion

Our study revealed that FTIR spectroscopy can detect changes in *S. aureus* WTA glycoepitope composition. A complete loss of *S. aureus* WTA expression resulted in strong perturbations in the spectral region assigned to carbohydrates and phosphorus-containing molecules. We successfully showed that FTIR spectroscopy is able to discriminate between α-/β-O-GlcNAc substitutions of *S. aureus* WTA. This novel approach complements the recently developed ANN-assisted FTIR spectroscopy capsular polysaccharide typing system and fosters the implementation of FTIR spectroscopy of whole bacterial cells for deciphering the *S. aureus* glycocode during colonization and the establishment and progression of staphylococcal infection.

## Methods

### Bacterial strains

RN4220 and NRS384 (US300) wild type and their isogenic mutant strains represent different glycosylation on WTA, which are listed in Table 2. A collection of well-characterized *S. aureus* strains (n = 70) from human, animal, and food sources was included in the study. Detailed information on all strains, including origin, clonal complex assignment, as well as comprehensive virulence and resistance gene profile determined by DNA microarray have been previously published^15^. All strains were analysed by PCR for the presence of the intact *tarM* locus according to Winstel *et al*.^20^.

**Table 2:** *S. aureus* strains used.

Strains	Glycosylation phenotype on WTA backbones	Source or reference
RN4220	WTA with predominant α-O-GlcNAc and β-O-GlcNAc glycoepitopes	Kreiswirth *et al*.^33^
RN4220 *ΔtarO*	Cell wall with no WTA	Xia *et al*.^8^
RN4220 *ΔtarM*	WTA with β-O-GlcNAc glycoepitopes	Brown *et al*.^7^
RN4220 *ΔtarS*	WTA with α-O-GlcNAc glycoepitopes	Brown *et al*.^7^
RN4220 *ΔtarM ΔtarS*	WTA with neither α-O-GlcNAc nor β-O-GlcNAc glycoepitopes	Brown *et al*.^7^
NRS384 (USA300)	Glycosylated WTA with both α-O-GlcNAc and β-O-GlcNAc glycoepitopes	NARSA collection
NRS384 *ΔtarO*	Cell wall with no WTA	Kurokawa *et al*.^23^
NRS384 *ΔtarM*	WTA with β-O-GlcNAc glycoepitopes	Kurokawa *et al*.^23^
NRS384 *ΔtarS*	WTA with α-O-GlcNAc glycoepitopes	Kurokawa *et al*.^23^
NRS384 *ΔtarM ΔtarS*	WTA with neither α-O-GlcNAc nor β-O-GlcNAc glycoepitopes	Kurokawa *et al*.^23^

### Whole cell FTIR spectroscopic measurements

FTIR spectroscopic measurements and spectral quality determination were performed as previously reported^15,21^. Duplicate measurements were performed on five different days. All strains were grown as a bacterial lawn on tryptone soy agar plates (Oxoid) at 30 °C. After 24 hours one loop-full of intact bacterial cells was suspended in 100 µl sterile deionized water. A 30 µL aliquot of the suspension was transferred to a zinc selenite (ZnSe) optical plate and dried at 40 °C for 40 min. Subsequently, FTIR spectra were recorded in transmission mode with an HTS-XT microplate adapter coupled to a Tensor 27 FTIR spectrometer (Bruker Optics GmbH, Ettlingen, Germany) using the following parameters: 4000–500 cm^−1^ spectral range, 6 cm^−1^ spectral resolution, zero-filling factor 4, Blackmann-Harris 3-term apodization, and 32 interferograms were averaged with background subtraction for each spectrum. Spectral quality assessment was performed for each spectrum with thresholds for minimum (0.300) and maximum (1.300) absorbance, signal to-noise (S/N) ratio, and water vapor content.

### Spectral processing and chemometrics

FTIR spectra were preprocessed as follows: (1) second derivatives were calculated over the whole spectral range using a second-order 9-point Savitzky–Golay algorithm, (2) spectra were vector normalized. Chemometric analysis was performed on preprocessed data employing unsupervised hierarchical cluster analysis (HCA) and principal component analysis (PCA). The spectral region between1200–800 cm^−1^ was selected for further analyses, as this region is highly discriminatory for *S. aureus* subtyping^15^ and is associated to polysaccharides and their specific types of glycosidic linkages (see also Table 1). For HCA, a dendrogram was generated using the average linkage algorithm at repro-level 30 using the OPUS software (Version 7.2; Bruker Optics GmbH). PCA computation was based on the NIPALS algorithm and the second and third components were projected for PCA using the software Unscrambler X (CAMO Software, Oslo, Norway).

### Cell wall extraction from *S. aureus* strains for FTIR spectroscopy

The *S. aureus* cell wall preparation was conducted according to Weidenmaier *et al*.^22^ and adapted for FTIR spectroscopic measurements. *S. aureus* cultures were centrifuged at 5000 × g for 10 min at RT and subsequently washed with 20 mM NH_4_Ac buffer (pH 4.8). After disruption in an Oneshot Frenchpress (Constant systems, Daventry, UK) using the same buffer, the cell lysates were centrifuged at 5000 × g for 10 min to remove potential intact cells. Next, SDS (final concentration of 2%) was added to the collected supernatant followed by ultra-sonication for 15 min to remove any cell membrane contaminations. After heating at 65 °C for one hour, the cell wall preparations were washed six times with 20 mM NH_4_Ac buffer by centrifugation at RT, 12000 × g for 10 minutes. Finally, the cell wall preparations were re-suspended in distilled water and measured by FTIR spectroscopy.

### Data availability

The datasets generated and/or analysed during the current study are available from the corresponding author on request.

## Electronic supplementary material

Supplementary Figures
