# Supplementary material for: Analysis of Staphylococcus aureus wall teichoic acid glycoepitopes by Fourier Transform Infrared Spectroscopy provides novel insights into the staphylococcal glycocode
Source: Sci Rep. 2018 Jan 30;8:1889. doi: 10.1038/s41598-018-20222-6 (PMC5789974; doi:10.1038/s41598-018-20222-6)
Supplement: Supplementary file 1 — Supplementary Figures [file 41598_2018_20222_MOESM1_ESM.pdf]

Supplementary Information

Analysis of *Staphylococcus aureus* wall teichoic acid glycoepitopes by Fourier Transform Infrared Spectroscopy provides novel insights into the staphylococcal glycocode

Tom Grunert[a,*], Dijana Jovanovic[a], Wanchat Sirisarn[b], Sophia Johler[c], Christopher Weidenmaier[d], Monika Ehling-Schulz[a], Guoqing Xia[b]

[a]Functional Microbiology, Institute of Microbiology, Department of Pathobiology, University of Veterinary Medicine Vienna, Vienna, Austria.

[b]Division of Infection, Immunity and Respiratory Medicine, School of Biological Sciences Faculty of Biology, Medicine and Health, Manchester Academic Health Science Centre , University of Manchester, Manchester, M13 9PT, United Kingdom.

[c]Institute for Food Safety and Hygiene, Vetsuisse Faculty University of Zurich, Zurich, Switzerland.

[d]Interfaculty Institute for Microbiology and Infection Medicine Tübingen, University of Tübingen and German Center for Infection Research, Tübingen, Germany.

Supplementary Fig. S1

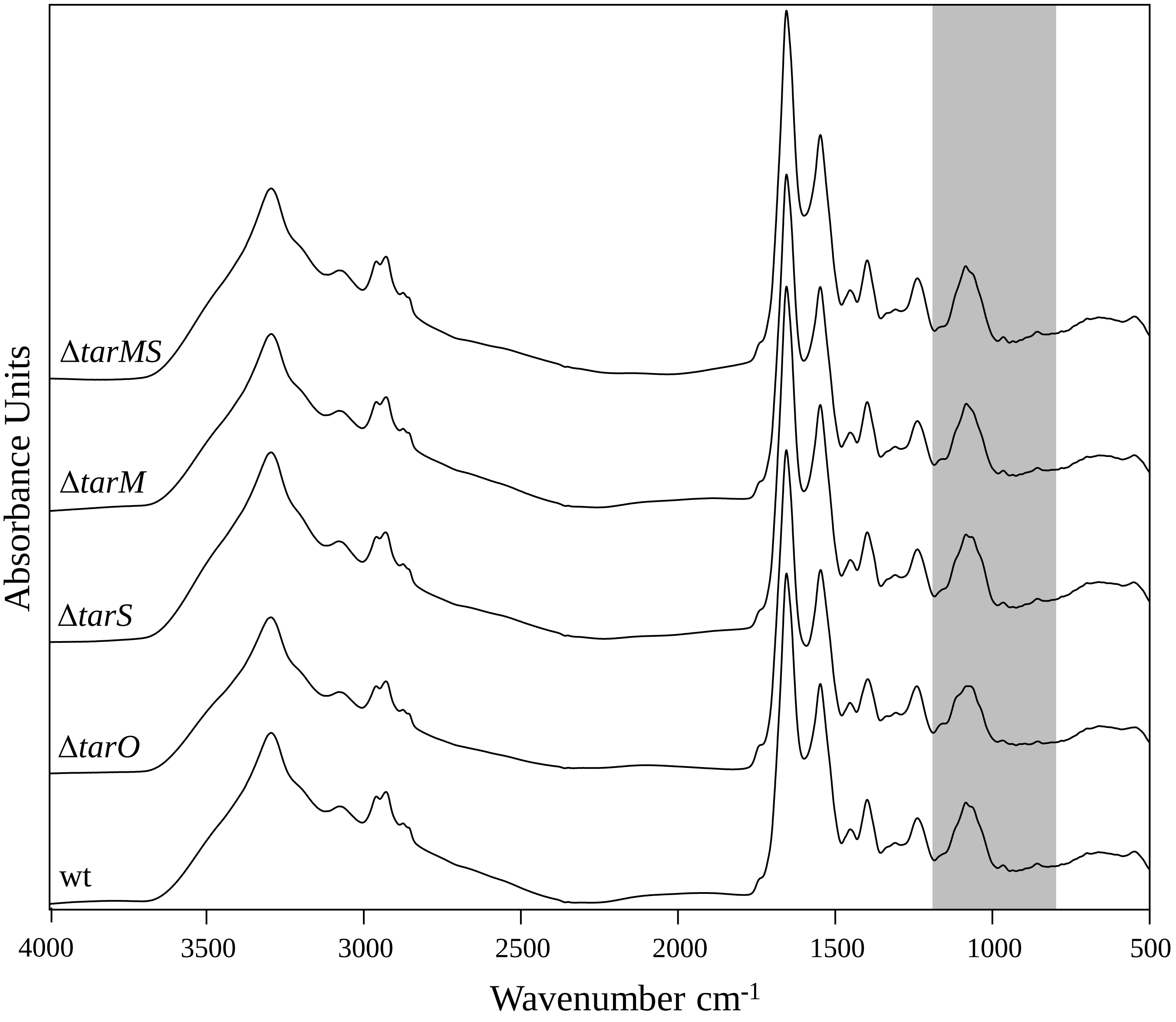

Representative original absorbance spectra over the whole spectral range (4000-500cm^{-1}) derived from intact bacterial cells of strain RN4220 and their corresponding mutants (Δ*tarO* , Δ*tarS,* Δ*tarM* and Δ*tarMS)*. The spectral range between 1200-800cm^{-1} (highlighted in gray), also refered as polysaccharide region, was used for further spectral and chemometric analysis.

Supplementary Fig. S2

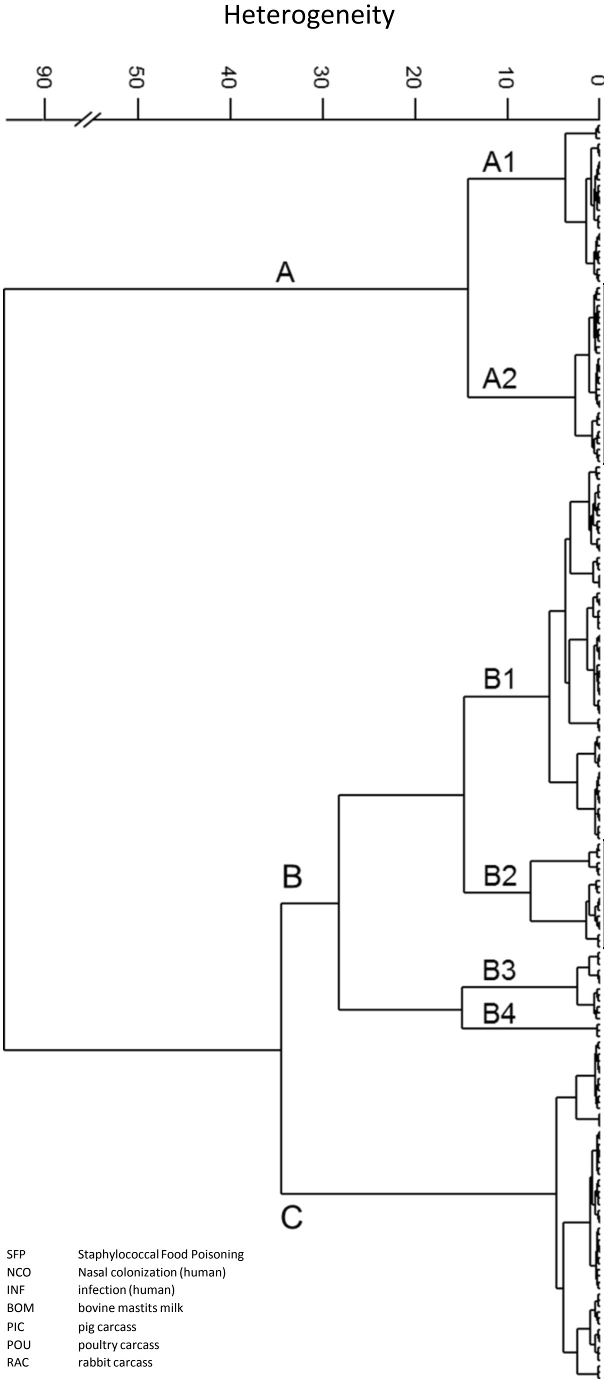

FTIR	*tarM*	ID	CP	*cap*	PFGE	CC	*spa*	*agr*	origin
A1A		1	8	8	5	45	t383	I	SFP
A1B		9	8	8	11	45	t1270	I	SFP
A1C		19	8	8	18	45	t630	I	NCO
A1C		22	8	8	20	45	t073	I	INF
A1C		25	8	8	23	45	t015	I	INF
A1C		27	8	8	25	45	t950	I	INF
A1C		31	8	8	28	45	t4460	I	INF
A1C		41	8	8	37	45	t050	I	NCO
A1C		68	8	8	55	45	t445	I	INF
A2A		10	8	8	12	151	t529	II	BOM
A2B		18	8	8	17	151	t529	II	BOM
A2B		30	8	8	3	151	t529	II	BOM
A2B		67	8	8	2	151	t529	II	BOM
A2C	+	23	8	8	21	151	t529	II	BOM
A2C		60	8	8	2	151	t529	II	BOM
A2C		70	8	8	3	151	t529	II	BOM
A2D		26	8	8	24	151	t529	II	BOM
A2E		54	8	8	3	151	t529	II	BOM
A2F		63	8	8	52	151	t529	II	BOM
B1A	+	2	NT	8	6	15	t4802	II	SFP
B1B	+	6	NT	5	9	8	t024	I	SFP
B1C		57	NT	5	49	5	t857	II	NCO
B1D	+	12	NT	5	13	8	t008	I	SFP
B1E		48	NT	5	42	5	t1062	II	NCO
B1F		44	5	8	39	5	t8020	I	NCO
B1G		62	8	8	51	8	t377	I	SFP
B1H		14	NT	5	15	9	t733	II	SFP
B1I		32	NT	5	NT	398	t2971	I	PIC
NT		28**	NT	5	26	22	t8019	I	PIC
B1J		58	NT	8	50	45	t1574	NT	NCO
B1K		29	NT	5	27	8	t148	I	INF
B1K		33	NT	5	29	5	t8017	II	NCO
B1L		53	NT	5	47	97	t359	I	NCO
B1M	+	59	NT	8	4	30	t017	III	POU
B1N		4	NT	8	8	12	t018	II	POU
B1O		66	NT	8	54	12	t209	II	INF
B1P		34	NT	5	30	97	t276	I	SFP
B1P	+	52	NT	5	46	8	t648	I	NCO
B1Q		50	NT	5	44	8	t148	I	SFP
B1R		65	NT	5	53	8	t008	I	SFP
B2A		8	NT	8	10	15	t279	II	RAC
B2B		40	NT	5	36	8	t681	I	SFP
B2C	+	15	NT	8	1	22	t1239	III	INF
B2D		37	NT	8	33	30	t018	III	NCO
B2E		51	NT	8	45	30	t8455	III	NCO
B2F		49	8	8	43	30	t4516	III	NCO
B3A		3	NT	8	7	30	t1076	III	NCO
B3B	+	7	NT	8	4	30	t012	III	NCO
B3C	+	43	HV	8	38	30	t021	III	NCO
B3D	+	47	NT	8	41	30	t021	III	INF
B4		24	NT	5	22	5	t002	II	PIC
C0A		5	5	5	NT	398	t4475	I	PIC
C0A		20	5	5	NT	398	t4475	I	PIC
C0A		56	5	5	NT	398	t034	I	NCO
C0B		45	5	5	NT	398	t571	I	PIC
C0C		64	5	5	NT	398	t034	I	PIC
C0D		11	5	5	NT	398	t2922	II	SFP
C0D		13	5	5	14	30	t018	III	SFP
C0D		16	5	5	1	30	t018	III	NCO
C0D	+	21	5	5	19	8	t334	I	RAC
C0E		39	5	5	35	5	t8456	II	INF
NT	+	17**	5	5	16	8	t008	I	RAC
C0F		36	5	5	32	5	t954	II	NCO
C0F		38	5	5	34	5	t179	II	INF
C0F		55	5	5	48	9	t209	II	NCO
C0G		35	5	5	31	5	t002	II	NCO
C0H		46	5	5	40	5	t010	II	PIC
C0I		42	5	5	NT	398	t011	I	PIC
C0J		61	5	5	NT	398	t034	I	INF
C0K		69	5	5	NT	398	t4475	I	INF

Correlation between the presence of the *tarM* gene and the strain specific signal signature of WTA α-O-GlcNAc analysed by HCA-FTIR spectroscopy using a diverse strain set of 70 isolates. It can be assumed that the presence of α-O-GlcNAc WTA may additionally contribute to the discrimination of *S. aureus* strains by FTIR spectroscopy, which is primarily based on CP expression (Cluster A: CP8; cluster B: NT; cluster C: CP8).

HCA was adapted from: Johler, S., Stephan, R., Althaus, D., Ehling-Schulz, M. & Grunert, T. High-resolution subtyping of *Staphylococcus aureus* strains by means of Fourier-transform infrared spectroscopy. *Syst. Appl. Microbiol.*, doi:10.1016/j.syapm.2016.03.003 (2016).
